# Urethan in Fever—Note from Dr. Beall

**Published:** 1887-07

**Authors:** E. J. Beall

**Affiliations:** Fort Worth, Texas


### URETHAN IN FEVER.

FORT WORTH, TEXAS, June 23, 1887.

*Dear Doctor Daniel*—A few days ago, I had under treatment a case of ataxic fever (typhoid). I had never seen such subsultus tendinum as existed in the case. The thermometer could not be kept in the mouth; the pulse could not be counted at the wrist, so great and constant did there occur muscular twitching. The symptoms present led me to prescribe the following medicine :

℞ Terebene - gtt xvj
Ac. Sulphurous gtt xl
Urethan - -
Antipyrine aa ℈i
Acacia Pd - q.s.
Elix. Aq. flor. Auranti ad ℥i
M Et Sig: One fourth part every 3 hours.

At the visit subsequent to the administration of the above, the chin and hands were as quiet as those of one in health.

On the succeeding, and for several days the same degree of subsultus obtained, and the administration of the same combination had the happy effect that was observed at its first administration.

What was the agent in the combination that controlled the ataxia? I think the urethan.

It is well known how little such unpleasant symptoms are controlled by the ordinary remedies hitherto used. If further observation shall corroborate the inferences here drawn, it is surely a point in therapy well worthy a niche in the brain of the practitioner of medicine.

E. J. BEALL.

